# Relative tail length correlates with body condition in male but not in female crowned leafnose snakes (*Lytorhynchus diadema*)

**DOI:** 10.1038/s41598-020-61168-y

**Published:** 2020-03-05

**Authors:** Jaim Sivan, Shlomo Hadad, Itay Tesler, Avi Rosenstrauch, Abraham Allan Degen, Michael Kam

**Affiliations:** 1 Department of Life Sciences, Achva Academic College, M. P. Shikmim, 79800 Israel; 2 Negev Zoo, P. O. B, 4033 Beer Sheva, Israel; 3 Desert Animal Adaptations and Husbandry, Wyler Department of Dryland Agriculture, Institutes for Desert Research, Ben Gurion University of the Negev, Beer Sheva, 8410500 Israel

**Keywords:** Behavioural ecology, Ecophysiology

## Abstract

Reproductive success is the ultimate measure of individual quality; however, it is difficult to determine in free-living animals. Therefore, indirect measures that are related to reproduction are generally employed. In snakes, males typically possess longer tails than females and this sexual size dimorphism in tail length (TL) has generally been attributed to the importance of the tail in mating and reproduction. Thus, intra-sexual differences in tail length, specifically within males, were hypothesized to reflect individual quality. We used a body condition index (BCI) as a measure of quality in snakes and predicted that tail length would be correlated with BCI in males. We tested our prediction by determining BCI in the free-ranging adult male and female crowned leafnose snake (*Lytorhynchus diadema*), a colubrid species that inhabits mainly desert sand dunes. The relative TL was correlated positively and significantly to BCI in males (F_1,131_ = 11.05; r^2^_adj_ = 0.07; *P* < 0.01) but not in females, thus supporting our prediction. This is the first time that the relationship between TL and body condition was tested in a free-ranging species. In addition, sexual size dimorphism of TL increased intra-specifically with body size, which was also found in interspecific analyses following Rensch’s rule.

## Introduction

Sexual size dimorphism (SSD) occurs in many species of invertebrate and vertebrate taxa^1,2^. In general, males are larger than females in bird and mammal species^3,4^, whereas females are larger than males in terrestrial vertebrate ectotherms^5,6^. According to Rensch’s rule, when males are the larger sex, SSD increases with size;^1,4^ however, when females are the larger sex, SSD decreases with size^1,7^. Although these trends seem contradictory, they both reflect greater variance in males than in females^8^. A number of hypotheses have been proposed to explain these evolutionary interspecific trends including sexual selection and mating success, fecundity selection favoring large females, and natural selection for resource partitioning^8–11^.

Sexual size dimorphism has also been observed in lengths of appendages such as in tails. In most snake species, males have relatively longer tails than females. The reason for this difference between sexes was initially explained structurally, as males possess a hemipenis in an elongated pocket at the base of the tail^12,13^. Several hypotheses for the evolution of sexual differences in tail length (TL) have been suggested. Interspecific analyses of tail and body lengths, as well as clutch mass in colubrid snakes, supported both the morphological constraint hypothesis and the female reproductive hypothesis. Consequently, these analyses indicated a male-biased TL dimorphism in taxa having relatively short tails^14^. In addition, the TL was found to be associated with gravitational habitat categories, where terrestrial snake species have shorter tails than arboreal species. This hypothesis was supported in females using 226 snake species in 139 genera and 15 families^15^.

Tails of snakes are important for a number of functions, including locomotion^12,15^, predation^16,17^ and reproduction^12,14,18^. However, a longer tail requires stronger retractor muscles to maneuver^14^ and, therefore, conforms to the power limits hypothesis^19^; that is, males with longer relative TL would be able to use more power over a short time for a successful mating and, consequently, have higher reproductive success.

A characteristic that is advantageous specifically for reproduction for one sex is considered a sexual selected trait. If TL of females is at the optimum size for hunting and locomotion^15^, then the additional length in males should serve a reproductive function, either to house a longer hemipenis or to increase mating opportunities^18^, and could be considered a sexual selected trait^20,21^. Relatively longer tails are advantageous when competing with other male snakes in the process of ball mating^18^ and, consequently, males with longer tails should be more productive^18^ and, thus, should demonstrate higher reproductive success than males with shorter tails. Males that are more successful are, consequently, of better quality. However, although commonly used, the term “individual quality” requires further clarification. Some authors have argued that it should be abandoned^22–24^, while others suggested using a more comprehensive approach such as multivariate analyses of selective traits^25^ or using a better defined term such as “genetic quality”^26^. Darwin^27^, Zahavi^28^ and Emlen *et al*.^21^, among others, related the term “individual quality” to the reproductive output or fitness of the individual. However, for most animal species, especially when free-ranging, it is not possible to determine individual reproductive success and, consequently, other indirect measures such as body mass, body size or body condition have often been used as individual quality indices/correlators (e.g., Hamel *et al*.^29^; Peig and Green^30^).

Body condition index (BCI) has been related to reproductive potential and reproductive success^31–34^, and, consequently, fitness of the animal^35–37^. BCI could be estimated using a non-invasive measure that is independent of body size and, in snakes, has been shown to reflect fat reserves^38^. This parameter has been described as being “intimately related to an animal’s health, quality or vigour and has been widely claimed to be an important determinant of fitness”^30^. In this study, we used BCI as a measure of individual quality, which we regarded as being related to reproductive success.

**Figure 1:**
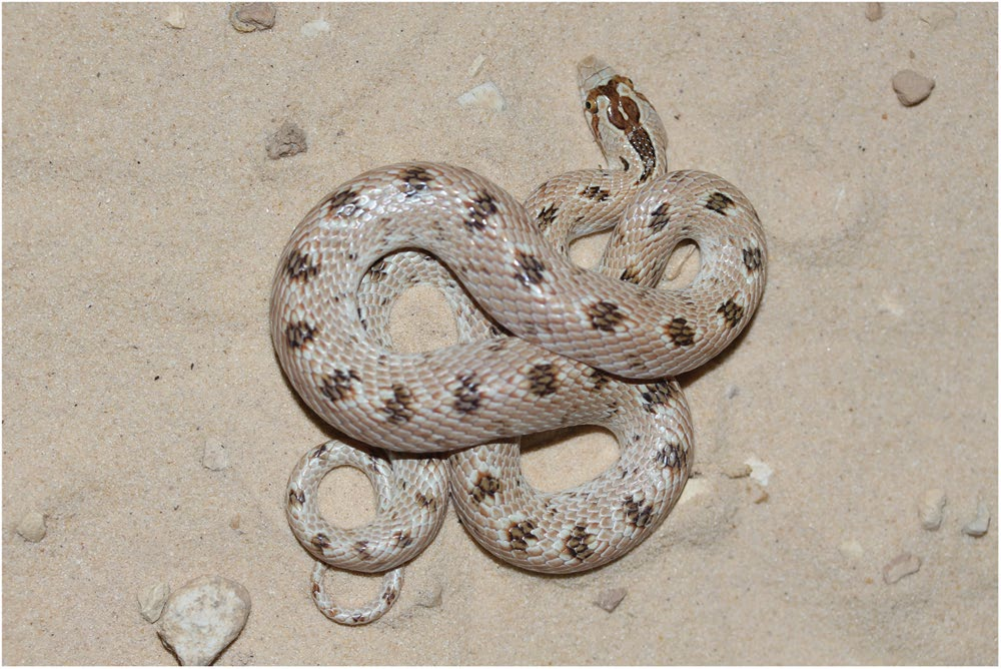
A *Lytorhynchus diadema* at our research site in the Negev Desert.

We hypothesized that TL is a sexual selective trait where males with relatively longer tails would be of higher individual quality. Using BCI to reflect individual quality, we predicted that males with longer tails would have a higher BCI than males with shorter tails, whereas, no such relationship would exist in females. We also predicted a higher variance of TL in males than in females as sexual characteristics often exhibit higher variability than non-sexual characteristics that are subject to evolutionary stabilizing selection^39,40^. To test our predictions, we determined BCI and TL in the free-ranging adult male and female crowned leafnose snake, *Lytorhynchus diadema* (Fig. 1). This colubrid species is a terrestrial dweller that possesses sexual dimorphism in both body length and TL and, therefore, is suitable to test our predictions^41^.

## Results

Average SVL of males (n = 133), 33.5 (±0.31) cm, was significantly longer than that of females (n = 107), 32.0 (±0.32) cm (F_1,238_ = 10.12, *P* < 0.01), the difference amounting to 4.5%; but the variance between sexes was similar (*P* = 0.348). Sexual size dimorphism in SVL was 1.047 and the compressed index was −0.047. Average TL in males, 5.91 (±0.068) cm, was significantly longer than in females, 5.28 (±0.058) (F_1,238_ = 45.61, *P* < 0.001), the difference amounting to 11.9%, and the variance in TL in males was 72% higher than in females (0.62 vs 0.36; *P* < 0.01). Tails of all males and females were intact.

**Figure 2:**
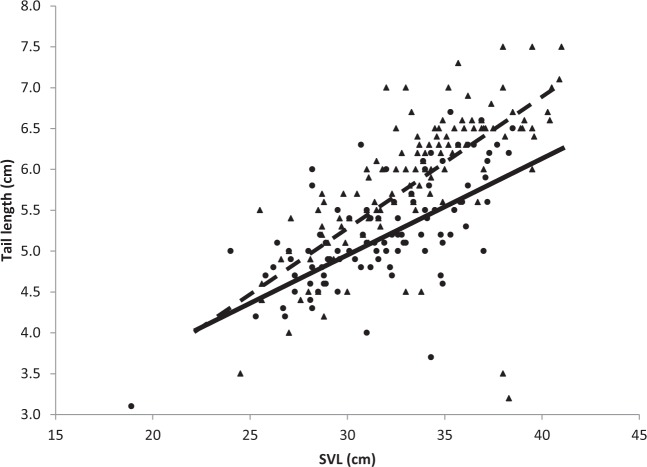
The effect of snout-vent length (SVL) on tail length in male (triangles; dashed line) and female (circles; solid line) *Lytorhynchus diadema*. The gap between the two regression lines represents the significant increase in sexual dimorphism of tail length with SVL; increase rate of tail length with SVL is higher in males than females (see text for explanation).

The regression of TL on SVL was significant in males (TL = 0.158 SVL + 0.573; r^2^ = 0.48; *P* < 0.01) and in females (TL = 0.118 SVL + 1.512; r^2^ = 0.44; *P* < 0.01), and explained more than 40% of the variation in TL in both sexes (Fig. 2); the slope of the regression equation in males was higher than that of females (S_b1-b2_ = 0.0190; t = 2.51; df = 236; *P* < 0.01). The proportion of TL to total length was 0.15 (±0.012) in males and 0.14 (±0.012) in females and the coefficient of divergence between sexes in TL was 5.5. Tail length dimorphism, based on the difference between sexes in the residuals of logTL (males = 0.0151; females = −0.0153), was 0.0304. Both size, as SVL (t = 14.62; *P* < 0.001), and sex (t = 6.14; *P* < 0.001) affected TL (F_(2, 237)_ = 150; r^2^_adj_ = 0.55; *P* < 0.001), and SSD of TL between sexes increased with size (Fig. 2).

**Figure 3:**
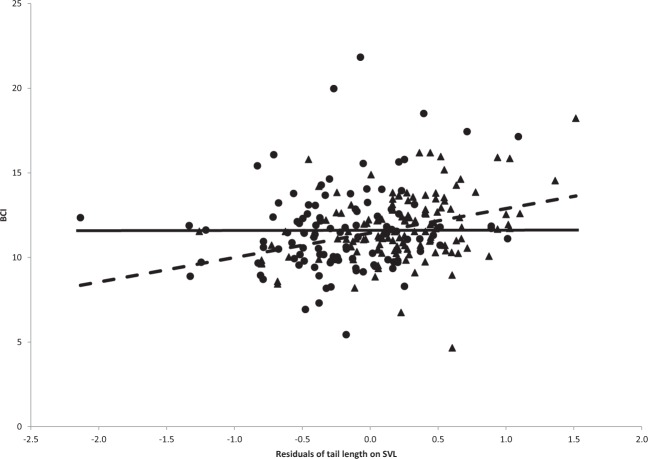
The effect of the residuals of tail length (TL_r_) on body condition index (BCI) in male (triangles; dashed line; the regression equation took the form of BCI = 1.01 TL_r_ + 11.73; r^2^ = 0.07; *P* < 0.01; n = 133) and female (circles and solid line at mean BCI because of non-significant relationship; *P* = 0.17; n = 107) free-ranging *Lytorhynchus diadema* (see text for explanation).

BCI did not differ (F_1,238_ = 0.381, *P* = 0.538) between males and females, 11.8 (±0.19) and 11.6 (±0.22), respectively. When data on TL from June to August were combined, a significant effect of resTL on BCI was found only in males (F_1,131_ = 11.05; r^2^_adj_ = 0.07; *P* < 0.01; Fig. 3). In males within each month, a significant effect of resTL on BCI was found in July (F_1,55_ = 5.31; r^2^_adj_ = 0.07; *P* < 0.05; n = 57) and in August (F_1,16_ = 15.15; r^2^_adj_ = 0.45; *P* < 0.01; n = 18) but not in June (F_1,56_ = 1.76; *P* = 0.19; n = 58), while, in females, no significant effect was found in any of the three months: June (F_1,42_ = 0.80, *P* = 0.38; n = 44), July (F_1,47_ = 1.43, *P* = 0.24; n = 49) and August (F_1,12_ = 0.21, *P* = 0.65; n = 14).

## Discussion

A significant effect of resTL on BCI emerged in males but not in females; consequently, our prediction was supported. The higher variance of TL in males than in females provides further support to our hypothesis that the tail of male *L. diadema* represents an intra-sexual trait. We questioned how could the non-significant effect for males in June be explained?

During the mating season, males invest much time in chasing and in mating females but not in feeding, as is common in a number of snake species^42^. Furthermore, in colubrid species, most of the body reserves are mobilized during reproductive activity, in particular in males^38,43^. In June, shortly after mating, male *L. diadema* were exhausted and that may explain the non-significant effect of resTL on BCI in this month. As the season progressed, males improved their body condition and in August, resTL explained 45% of the variance in BCI. However, this interpretation should be taken cautiously as BCI varies temporally and can change among years depending on environmental conditions.

### Tails as sexual traits in males

Most hypotheses examining TL were tested interspecifically, where a mean value for each species was taken. We are aware of only one intraspecific study in snakes^18^ and, in that study, the hemipenis length of the male garter snake (*Thamnophis sirtalis parietalis*) was correlated positively with the residuals of TL on snout-vent length (SVL) and, apparently, supported the morphological constraint hypothesis. However, SVL was used to correct only for TL (as residuals), and not for hemipenis length. In addition, this study concluded, albeit indirectly, that the tail was important in mating, as males with a partial tail loss had a lower mating success than males with an intact tail. When males with intact tails were tested, individuals with longer TL had higher mating success, but that “could simply reflect the mating advantage accruing to larger overall body size”. When relative TL using residuals of TL on SVL was compared in mated versus unmated males, no difference in relative TL was found^18^.

Based on our findings, we suggest a novel perspective on the length and function of the tail in male *L. diadema*, which could be applicable to other snake species. We found that the residuals of TL on body size in *L. diadema* were significantly and positively correlated with BCI in males but not in females. Interpreting these data from a different perspective, males with longer tails are compensated for the extra energetic cost of supporting their heavier tails; the distance to the regression line (Fig. 3) indicates the relative quality of each snake^44^. Following the handicap hypothesis, an “honest signal” reflects individual quality^28,45^. Longer tails may house longer hemipenes, but that in itself may not be sufficient for successful mating, as was evident for individuals with cut tails^18^. However, longer tails utilize more energy and, during ball mating, require stronger retractor muscles to maneuver and compete successfully with other potential mates. In many cases, handicapped appendages are sexually selected ornaments used by males for display during courtship to attract female mates (see review of Clark^19^). Here, we relate to “handicap” in a broader viewpoint. The tail of male *L. diadema* is not used for display but rather as an intra-sexual selected trait^46^ or an “intrinsically unfakable index signal”^21^ and, indeed, has shown to be more variable in males than females. In addition, compensatory traits are expected to cover the additional costs of the sexual character, tail length in our study, which, in turn, increase the probability of successful mating with a female (e.g. review by Husak and Swallow^44^).

Body condition is an important parameter affecting both fecundity and fitness and is a measure of body energy reserves and, hence, differentiates among individuals on the basis of their quality. Here, and for the first time, we propose an intraspecific link between a specific characteristic and individual quality in free-ranging snakes. Furthermore, we were able to statistically explain much of the variance in TL and suggest consequences of individual quality.

### Sexual dimorphism

In most snake species, females are longer (in SVL) than males and in 56 colubrid genera, 73% conformed to this SSD^14^. Within the group of longer males than females, SSD ranged between 1.010 in *Cyclocorus* and 1.498 in *Drymoluber*^14^. In snakes, body size of the female is related with its reproductive effort and, consequently, with mode of reproduction. The “female reproductive output hypothesis” implies that SSD should be more female-biased in oviparous taxa than in viviparous taxa^14^. Interestingly, all 15 colubrid genera with longer males than females were oviparous. *L. diadema* fell within this minority group with a SVL dimorphism of 1.047. Furthermore, females within this group exhibited relatively longer tails than males except for one genus, *Cyclocorus*, with a TLD of 0.029, which was similar to the 0.030 of *L. diadema*. Within oviparous colubrids, females had relatively longer tails than males in more than 70% (29 of 41) of the genera (calculated from King^14^).

The proportion of TL to total length of adult *L. diadema* females (0.14 ± 0.012) was similar to the average for 49 non-scansorial colubrid female snakes^15^ (0.17 ± 0.060) and was substantially lower than the average for arboreal colubrid female snakes (0.31 ± 0.073). Consequently, the sexual dimorphism in TL was higher than that of arboreal colubrid snake species. The coefficient of divergence in TL of 5.5 in *L. diadema* was at the lower end of the range for 49 colubrid snakes^13^. However, the TL dimorphism index, which is a more balanced index and independent of body size, was higher in *L. diadema* than that of many other colubrid snake species^14^, and fell in the upper 25% of values. We conclude that the high TL dimorphism index in *L. diadema* reflects the importance of the male TL for successful mating.

### Consequences of rensch’s rule

Rensch’s rule states that SSD typically increases with body size in interspecific comparisons when males are the larger sex. There have been a number of hypotheses explaining this generally accepted evolutionary trend in animals^8,11,47,48^, including in snakes^10,49,50^. It was also stated that males would exhibit greater variance in size than females^8^. In *L. diadema*, the male is the larger sex and the variance in TL was higher than that in the female. Our study design allowed testing whether evolutionary perspectives of Rensch’s rule can be applied intra-specifically as well as interspecifically for sexual dimorphism in TL. Our analysis indicated that it can, as SSD of TL between sexes increased with body size. It would be interesting to examine similar intraspecific relationships across various colubrid species and other taxa to test whether these findings reflect a general trend.

## Methods

### Snake species and study site

**Figure 4:**
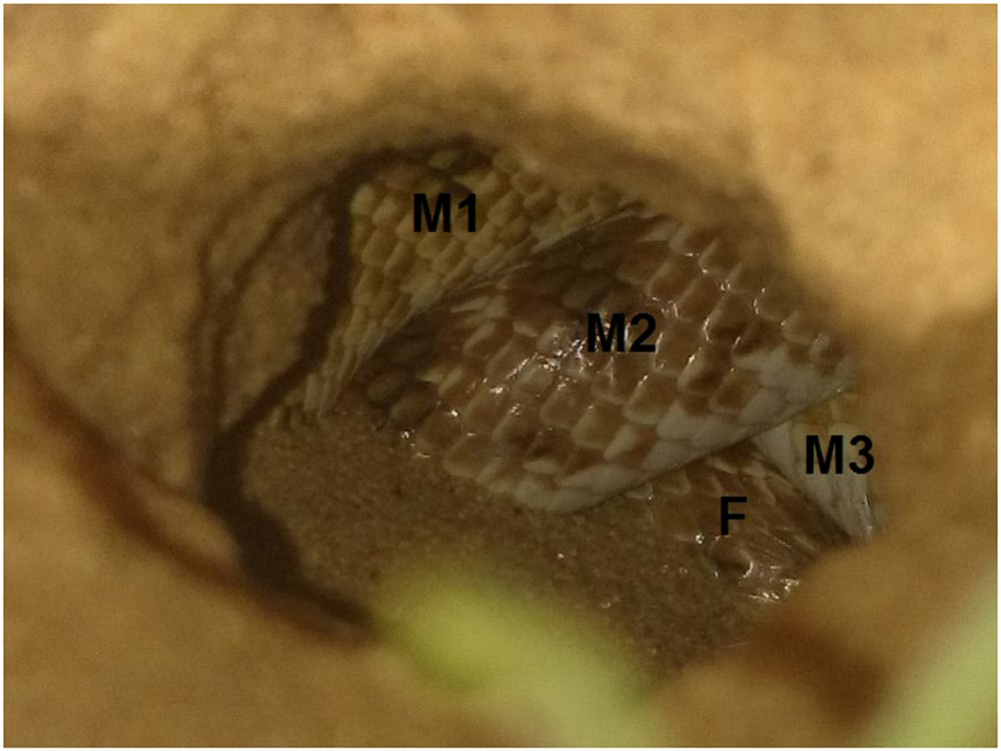
Three *Lytorhynchus diadema* males (M1, M2, M3) coiled around a female (F) at the entrance of a rodent burrow in a ‘mating ball’. *L. diadema* vary in color allowing the distinction among individuals.

The crowned leafnose (*Lytorhynchus diadema*) inhabits mainly desert dunes ranging from the Atlantic coast to the Red Sea in North Africa, northward to the Mediterranean coast and eastward to the Arava (part of the Rift Valley). It also occurs allopathically in other deserts and along the Mediterranean coast of Israel^51^. This colubrid species is active from April to October and is not active in winter^52^. It is a nocturnal hunter consuming primarily small lizards and lizard eggs^52^. Mating occurs in April - May^53^ when “mating balls” are formed. In this reproductive process, several males are coiled around a female (Fig. 4), as has been documented in other colubrid snakes^18^. Females lay 3–5 eggs in the sand in June - July^53^ and young hatch synchronously in August^41^. During the current study, we observed three events of egg laying, all in late June. The three females were not handled due to nature protection restrictions and, therefore, were not included in the analyses.

The study was undertaken in the desert sand dunes surrounding *Wadi Seher*, situated in the western Negev, Israel, 15 km south of Beer-Sheva (31° 5.4ʹ N and 34° 48.6ʹ E), at 320 to 340 m above sea level. Annual rainfall at the site averages 150 mm, and is characterized by high annual variations and in temporal and spatial distribution. All rainfall occurs in winter from November to March with 60% generally falling in December and January, and, consequently, other than dew, no surface water was available to the snakes during the entire study. Winters are mild; the coldest month, January, has mean minimum and maximum air temperatures of 7.6 °C and 18.1 °C, respectively. Summers are hot and dry lasting from June to October. The hottest month, August, has mean minimum and maximum air temperatures of 20.2 °C and 33.5 °C, respectively^54^. The dominant vegetation is of the Saharo-Arabian type with the main plants consisting of *Artemisia monosperma*, *Stipagrostis scoparia*, *Convolvulus lanatus*, *Retama raetam*, *Stipa capersis*, *Hammada scoparia*, *Neurada procumbes* and *Noaea mucronata*.

### Capture and measurements

Random scans of the area were made on foot from dawn to dusk to identify the typical lateral weaving serpentine tracks of *L. diadema*^41,52^. Tracks were followed and the snakes were captured by hand. The sex of the snake was based on the distinct difference between males and females in the depth of the sub-caudal pocket^55,56^. A reptile sexing probe was inserted gently into the cloaca towards the tip of the tail to determine the number of sub-caudals; females measured 1–3 whereas males measured > 4. Snakes were weighed to the nearest 0.01 g (Ohaus Balance, Model CT 200) and SVL and TL were measured to the nearest 0.1 cm using a measuring tape after securing the snake gently on a board. To minimize errors, two researchers (JS and SH) made all measurements. Snakes were handled for less than 5 min to complete the measurements and then were released at the place of capture. On the bases of SVL and month of capture, *L. diadema* were classified as neonates, juveniles and adults^41^; only adults were included in this study. Re-capture of the same individual at the study site is low; based on marked individuals at the same site in a previous study, less than 5% were re-captured within the same month (unpublished data). Hence, for statistical analyses, each measurement was considered independent. Data were collected over 5 years on 162 nights in June, July and August when no rain fell and, in total, 133 males and 107 females were used in the analyses (Supplementary Table 1). All procedures on the snakes were approved by the Israel Nature and National Parks Protection Authority and the Ben-Gurion University Committee for the Ethical Care and Use of Animals in Experiments. All methods were carried out in accordance with relevant guidelines and regulations.

### Body condition index and sexual dimorphism

In snakes, BCI is commonly estimated from the residuals of the ordinary least squares (OLS) intraspecific regression of body mass on SVL^35,36,57,58^, where SVL is taken as the measure of body size^59^. Using this method, approximately 50% of the variation in BCI was found to be due to variation in fat bodies, which allowed this method to test for differences in body condition statistically^60^. In a laboratory study, when BCI was corrected for ingesta (or recent feed intake), there was little difference in the estimation of fat, which implied that BCI can be measured in free-living snakes “without having to allow the snakes to clear their digestive tracts”^60^. To estimate BCI in the present study, we used the “scaled mass index”, which has been shown to be even a better predictor of variations in fat and protein reserves than the OLS residual method and five other methods^30,61^. Briefly, this index is based on the reduced major axis (RMA) method for calculating a structural relationship^62^. The RMA method, which is appropriate for our study species (see Smith^63^), allows errors in both variables and both are not affected by scale transformations. Controversy has arisen recently regarding whether RMA or OLS regression is most appropriate for studies of allometry and scaling, and OLS was suggested as a preferred statistical method (e.g. Kilmer and Rodríguez^64^). However, for calculating BCI, the aim of the regression is not to predict the body mass of a non-measured individual given its SVL, but to estimate the scaling exponent between two interdependent variables^50,65^. Further support and a thorough review, justification and validation of this method were presented by Peig and Green^61^; calculation of the BCI of free-ranging *L. diadema*^41^ followed their scaled mass index. Neither surface water nor rainfall was available at the study site when the snakes were active. However, dew was available and, therefore, dehydration should not have been a factor in using BCI as a measure of body condition in this study.

Sexual size dimorphism was calculated following Lovich and Gibbons^66^ (“compressed” SDI) as [SVL of the larger sex/SVL of the smaller sex minus 1]; by convention, negative when males are the larger sex and positive when females are the larger sex. In this index, values are symmetric around zero. To allow for comparison with other snake species, we also present sexual dimorphism in snout-vent length (SVLD) following King^14^. Dimorphism in tail length (TLD) was calculated from the regression of TL on SVL as the difference in mean residual TL between sexes^14^. The data underwent log transformations to allow for interspecific comparisons^14^ and the “coefficient of divergence” in tail length between sexes was calculated to allow for comparison with the data of Clark^13^.

### Statistics

To remove the effect of SVL (size) on TL and to allow for comparisons among different sized individuals, residual TL (resTL) was calculated separately for each sex^14^. A t-test was used to test for differences in SVL, TL and BCI between sexes, and the Levene test was used to test for differences in variance. To examine the effect of SSD of TL on SVL intra-specifically, an ANCOVA was used to test for difference between the two regression estimates of TL on SVL in males and females. Measurements are presented as means (±SE) and *P* < 0.05 was accepted as the level of significance. Statistica 7 (Stat Soft Inc., Tulsa, OK, USA) was used for statistical analyses.

## Supplementary information

Supplementary information
