# Supplementary material for: Relative tail length correlates with body condition in male but not in female crowned leafnose snakes (Lytorhynchus diadema)
Source: Sci Rep. 2020 Mar 5;10:4130. doi: 10.1038/s41598-020-61168-y (PMC7058055; doi:10.1038/s41598-020-61168-y)
Supplement: Supplementary file 1 — Supplementary information [file 41598_2020_61168_MOESM1_ESM.docx]

Supplementary Table for:

Relative tail length correlates with body condition in male but not in female crowned leafnose snakes (*Lytorhynchus* *diadema*)

Jaim Sivan, Shlomo Hadad, Itay Tesler, Avi Rosenstrauch, Abraham Allan Degen and Michael Kam

Supplementary Table 1.

Snout-vent length (SVL; cm), tail length (Tail; cm) and body condition index (BCI) of free-ranging adult female and male crowned leafnose (*Lytorhynchus diadema*) snakes in June, July and August. Each row represents a different individual.

| **Month** | **Sex** | **SVL** | **BCI** | **Tail** |
| --- | --- | --- | --- | --- |
| June | f | 34.3 | 12.35 | 3.7 |
| June | f | 31.0 | 11.88 | 4.0 |
| June | f | 26.7 | 10.53 | 4.3 |
| June | f | 27.3 | 10.05 | 4.5 |
| June | f | 28.1 | 11.71 | 4.6 |
| June | f | 27.3 | 21.83 | 4.7 |
| June | f | 32.3 | 15.41 | 4.7 |
| June | f | 26.2 | 11.55 | 4.8 |
| June | f | 32.2 | 12.38 | 4.8 |
| June | f | 29.0 | 9.93 | 4.9 |
| June | f | 31.6 | 9.55 | 4.9 |
| June | f | 29.6 | 11.49 | 4.9 |
| June | f | 27.0 | 11.06 | 5.0 |
| June | f | 32.0 | 9.79 | 5.0 |
| June | f | 32.0 | 11.43 | 5.0 |
| June | f | 37.0 | 9.72 | 5.0 |
| June | f | 31.0 | 9.84 | 5.1 |
| June | f | 31.9 | 12.34 | 5.1 |
| June | f | 33.0 | 12.12 | 5.1 |
| June | f | 30.8 | 11.89 | 5.2 |
| June | f | 33.8 | 9.93 | 5.2 |
| June | f | 32.3 | 13.67 | 5.2 |
| June | f | 34.8 | 16.07 | 5.2 |
| June | f | 30.1 | 9.71 | 5.4 |
| June | f | 31.2 | 9.47 | 5.4 |
| June | f | 31.6 | 14.05 | 5.4 |
| June | f | 34.1 | 13.07 | 5.4 |
| June | f | 34.0 | 8.26 | 5.5 |
| June | f | 34.0 | 9.77 | 5.5 |
| June | f | 35.5 | 10.17 | 5.5 |
| June | f | 34.5 | 12.16 | 5.5 |
| June | f | 35.8 | 12.57 | 5.6 |
| June | f | 37.2 | 13.22 | 5.6 |
| June | f | 33.3 | 10.24 | 5.7 |
| June | f | 34.2 | 13.25 | 5.8 |
| June | f | 37.1 | 14.28 | 5.9 |
| June | f | 32.0 | 11.79 | 6.0 |
| June | f | 34.0 | 12.57 | 6.0 |
| June | f | 33.9 | 13.13 | 6.1 |
| June | f | 37.3 | 11.21 | 6.2 |
| June | f | 30.7 | 11.11 | 6.3 |
| June | f | 35.7 | 15.79 | 6.3 |
| June | f | 37.7 | 15.55 | 6.3 |
| June | f | 35.3 | 17.44 | 6.7 |
| July | f | 26.8 | 12.30 | 4.2 |
| July | f | 25.3 | 19.98 | 4.2 |
| July | f | 28.5 | 13.10 | 4.5 |
| July | f | 28.9 | 11.20 | 4.6 |
| July | f | 28.8 | 11.90 | 4.6 |
| July | f | 34.9 | 8.89 | 4.6 |
| July | f | 25.8 | 12.84 | 4.7 |
| July | f | 28.7 | 11.77 | 4.8 |
| July | f | 31.2 | 13.78 | 4.8 |
| July | f | 27.1 | 11.66 | 4.9 |
| July | f | 30.4 | 10.17 | 4.9 |
| July | f | 29.1 | 13.77 | 4.9 |
| July | f | 29.5 | 9.36 | 5.0 |
| July | f | 32.6 | 10.87 | 5.0 |
| July | f | 26.4 | 11.31 | 5.1 |
| July | f | 31.4 | 9.69 | 5.1 |
| July | f | 31.9 | 10.18 | 5.1 |
| July | f | 34.9 | 9.66 | 5.1 |
| July | f | 32.9 | 12.01 | 5.1 |
| July | f | 30.8 | 9.22 | 5.2 |
| July | f | 32.6 | 8.92 | 5.2 |
| July | f | 28.6 | 13.95 | 5.2 |
| July | f | 32.8 | 11.36 | 5.2 |
| July | f | 35.3 | 10.60 | 5.2 |
| July | f | 35.3 | 10.94 | 5.2 |
| July | f | 36.1 | 8.94 | 5.3 |
| July | f | 32.6 | 5.44 | 5.4 |
| July | f | 30.1 | 9.81 | 5.4 |
| July | f | 32.6 | 10.62 | 5.4 |
| July | f | 31.0 | 9.35 | 5.5 |
| July | f | 29.5 | 18.50 | 5.5 |
| July | f | 35.9 | 6.92 | 5.6 |
| July | f | 32.4 | 12.44 | 5.6 |
| July | f | 33.4 | 12.72 | 5.6 |
| July | f | 37.2 | 10.50 | 5.6 |
| July | f | 28.2 | 11.72 | 5.8 |
| July | f | 28.2 | 11.85 | 5.8 |
| July | f | 36.2 | 8.17 | 5.8 |
| July | f | 32.0 | 10.72 | 6.0 |
| July | f | 28.2 | 17.14 | 6.0 |
| July | f | 34.0 | 15.64 | 6.0 |
| July | f | 34.9 | 9.74 | 6.1 |
| July | f | 37.2 | 10.50 | 6.1 |
| July | f | 34.3 | 11.09 | 6.2 |
| July | f | 38.3 | 10.02 | 6.2 |
| July | f | 35.7 | 8.29 | 6.3 |
| July | f | 37.7 | 9.15 | 6.3 |
| July | f | 38.5 | 9.56 | 6.5 |
| July | f | 36.9 | 10.37 | 6.6 |
| August | f | 28.2 | 11.47 | 4.3 |
| August | f | 28.1 | 10.56 | 4.4 |
| August | f | 28.8 | 14.64 | 4.7 |
| August | f | 34.8 | 11.62 | 4.7 |
| August | f | 28.2 | 12.85 | 4.8 |
| August | f | 30.7 | 11.47 | 4.8 |
| August | f | 28.0 | 11.43 | 5.0 |
| August | f | 28.0 | 11.63 | 5.0 |
| August | f | 28.0 | 11.82 | 5.0 |
| August | f | 31.5 | 9.42 | 5.0 |
| August | f | 34.0 | 8.71 | 5.0 |
| August | f | 28.9 | 14.03 | 5.1 |
| August | f | 32.6 | 7.30 | 5.2 |
| August | f | 36.5 | 9.87 | 6.3 |
| June | m | 38.3 | 11.83 | 3.2 |
| June | m | 28.0 | 14.20 | 4.5 |
| June | m | 28.5 | 15.79 | 4.5 |
| June | m | 28.1 | 14.89 | 4.9 |
| June | m | 29.0 | 10.77 | 5.1 |
| June | m | 28.9 | 12.26 | 5.1 |
| June | m | 31.1 | 12.65 | 5.1 |
| June | m | 28.7 | 11.17 | 5.2 |
| June | m | 29.6 | 13.36 | 5.4 |
| June | m | 31.7 | 9.35 | 5.5 |
| June | m | 28.5 | 15.18 | 5.5 |
| June | m | 31.0 | 13.45 | 5.5 |
| June | m | 33.5 | 12.62 | 5.5 |
| June | m | 33.4 | 10.34 | 5.6 |
| June | m | 36.7 | 10.03 | 5.6 |
| June | m | 28.7 | 10.55 | 5.7 |
| June | m | 30.5 | 16.19 | 5.7 |
| June | m | 34.3 | 11.17 | 5.9 |
| June | m | 32.5 | 10.96 | 6.0 |
| June | m | 33.5 | 10.17 | 6.0 |
| June | m | 31.8 | 13.54 | 6.0 |
| June | m | 31.0 | 14.63 | 6.0 |
| June | m | 37.0 | 9.88 | 6.0 |
| June | m | 34.3 | 13.83 | 6.0 |
| June | m | 37.0 | 13.84 | 6.0 |
| June | m | 33.9 | 11.53 | 6.1 |
| June | m | 32.8 | 12.86 | 6.2 |
| June | m | 33.6 | 12.05 | 6.2 |
| June | m | 35.2 | 9.10 | 6.3 |
| June | m | 36.1 | 10.23 | 6.3 |
| June | m | 35.7 | 11.28 | 6.3 |
| June | m | 34.7 | 12.61 | 6.3 |
| June | m | 38.1 | 12.22 | 6.4 |
| June | m | 34.6 | 15.96 | 6.4 |
| June | m | 39.6 | 11.10 | 6.4 |
| June | m | 34.7 | 4.66 | 6.5 |
| June | m | 32.5 | 11.67 | 6.5 |
| June | m | 37.5 | 10.41 | 6.5 |
| June | m | 37.1 | 10.87 | 6.5 |
| June | m | 32.5 | 15.91 | 6.5 |
| June | m | 36.5 | 12.18 | 6.5 |
| June | m | 36.0 | 12.84 | 6.5 |
| June | m | 37.5 | 11.52 | 6.5 |
| June | m | 35.5 | 13.47 | 6.5 |
| June | m | 35.5 | 13.81 | 6.5 |
| June | m | 36.0 | 13.68 | 6.5 |
| June | m | 38.9 | 11.09 | 6.5 |
| June | m | 36.9 | 10.73 | 6.6 |
| June | m | 40.4 | 11.53 | 6.6 |
| June | m | 38.5 | 6.75 | 6.7 |
| June | m | 37.4 | 12.68 | 6.8 |
| June | m | 36.2 | 13.86 | 6.9 |
| June | m | 38.0 | 8.95 | 7.0 |
| June | m | 35.2 | 11.72 | 7.0 |
| June | m | 33.0 | 14.54 | 7.0 |
| June | m | 32.0 | 18.22 | 7.0 |
| June | m | 39.5 | 10.07 | 7.5 |
| June | m | 38.0 | 12.59 | 7.5 |
| July | m | 28.8 | 9.61 | 4.2 |
| July | m | 28.8 | 9.67 | 4.2 |
| July | m | 28.8 | 9.84 | 4.2 |
| July | m | 25.6 | 8.20 | 4.4 |
| July | m | 27.6 | 11.36 | 4.4 |
| July | m | 28.0 | 9.89 | 4.5 |
| July | m | 33.8 | 11.55 | 4.5 |
| July | m | 26.6 | 12.41 | 4.9 |
| July | m | 29.3 | 11.07 | 4.9 |
| July | m | 27.0 | 10.50 | 5.0 |
| July | m | 28.9 | 10.26 | 5.1 |
| July | m | 30.8 | 11.10 | 5.2 |
| July | m | 28.7 | 13.57 | 5.3 |
| July | m | 31.7 | 10.63 | 5.3 |
| July | m | 29.7 | 13.13 | 5.3 |
| July | m | 31.7 | 13.11 | 5.3 |
| July | m | 30.1 | 10.45 | 5.4 |
| July | m | 29.6 | 12.48 | 5.4 |
| July | m | 31.6 | 12.02 | 5.4 |
| July | m | 31.5 | 11.12 | 5.5 |
| July | m | 31.5 | 11.39 | 5.5 |
| July | m | 28.8 | 10.29 | 5.6 |
| July | m | 32.4 | 10.45 | 5.6 |
| July | m | 34.9 | 12.20 | 5.6 |
| July | m | 29.8 | 13.34 | 5.7 |
| July | m | 32.3 | 12.80 | 5.7 |
| July | m | 34.3 | 10.95 | 5.7 |
| July | m | 33.2 | 11.15 | 5.8 |
| July | m | 31.1 | 10.72 | 5.9 |
| July | m | 35.0 | 9.52 | 6.0 |
| July | m | 35.0 | 10.50 | 6.0 |
| July | m | 35.0 | 11.27 | 6.0 |
| July | m | 34.0 | 13.63 | 6.0 |
| July | m | 33.0 | 16.18 | 6.0 |
| July | m | 39.5 | 11.53 | 6.0 |
| July | m | 34.0 | 12.57 | 6.2 |
| July | m | 34.8 | 12.35 | 6.2 |
| July | m | 33.7 | 10.40 | 6.3 |
| July | m | 34.7 | 9.75 | 6.3 |
| July | m | 34.2 | 10.29 | 6.3 |
| July | m | 34.7 | 12.68 | 6.3 |
| July | m | 33.6 | 11.11 | 6.4 |
| July | m | 35.1 | 11.04 | 6.4 |
| July | m | 34.5 | 10.99 | 6.5 |
| July | m | 36.0 | 11.54 | 6.5 |
| July | m | 36.0 | 12.64 | 6.5 |
| July | m | 34.5 | 14.27 | 6.5 |
| July | m | 37.0 | 12.15 | 6.5 |
| July | m | 37.0 | 12.21 | 6.5 |
| July | m | 39.0 | 11.05 | 6.5 |
| July | m | 36.9 | 13.82 | 6.5 |
| July | m | 39.1 | 8.85 | 6.6 |
| July | m | 36.4 | 11.20 | 6.6 |
| July | m | 35.9 | 12.93 | 6.6 |
| July | m | 33.3 | 11.94 | 6.7 |
| July | m | 40.3 | 11.98 | 6.7 |
| July | m | 40.9 | 9.85 | 7.1 |
| August | m | 27.0 | 10.72 | 4.0 |
| August | m | 30.0 | 8.43 | 4.5 |
| August | m | 30.0 | 8.58 | 4.5 |
| August | m | 29.7 | 12.80 | 5.3 |
| August | m | 27.1 | 12.36 | 5.4 |
| August | m | 31.1 | 12.16 | 5.4 |
| August | m | 25.5 | 12.55 | 5.5 |
| August | m | 31.0 | 11.01 | 5.5 |
| August | m | 31.4 | 10.53 | 5.6 |
| August | m | 33.7 | 12.18 | 5.8 |
| August | m | 34.0 | 13.67 | 6.0 |
| August | m | 35.1 | 11.95 | 6.4 |
| August | m | 35.5 | 11.70 | 6.5 |
| August | m | 37.0 | 10.71 | 6.5 |
| August | m | 39.5 | 9.87 | 6.5 |
| August | m | 34.9 | 11.78 | 6.6 |
| August | m | 35.2 | 15.85 | 7.0 |
| August | m | 41.0 | 10.24 | 7.5 |
